# Nucleus Accumbens Shell and mPFC but Not Insula Orexin-1 Receptors Promote Excessive Alcohol Drinking

**DOI:** 10.3389/fnins.2016.00400

**Published:** 2016-08-30

**Authors:** Kelly Lei, Scott A. Wegner, Ji Hwan Yu, Arisa Mototake, Bing Hu, Frederic W. Hopf

**Affiliations:** Alcohol and Addiction Research Group, Department of Neurology, University of California, San FranciscoSan Francisco, CA, USA

**Keywords:** alcohol, nucleus accumbens shell, insula, orexin, SB-334867

## Abstract

Addiction to alcohol remains a major social and economic problem, in part because of the high motivation for alcohol that humans exhibit and the hazardous binge intake this promotes. Orexin-1-type receptors (OX1Rs) promote reward intake under conditions of strong drives for reward, including excessive alcohol intake. While systemic modulation of OX1Rs can alter alcohol drinking, the brain regions that mediate this OX1R enhancement of excessive drinking remain unknown. Given the importance of the nucleus accumbens (NAc) and anterior insular cortex (aINS) in driving many addictive behaviors, including OX1Rs within these regions, we examined the importance of OX1Rs in these regions on excessive alcohol drinking in C57BL/6 mice during limited-access alcohol drinking in the dark cycle. Inhibition of OX1Rs with the widely used SB-334867 within the medial NAc Shell (mNAsh) significantly reduced drinking of alcohol, with no effect on saccharin intake, and no effect on alcohol consumption when infused above the mNAsh. In contrast, intra-mNAsh infusion of the orexin-2 receptor TCS-OX2-29 had no impact on alcohol drinking. In addition, OX1R inhibition within the aINS had no effect on excessive drinking, which was surprising given the importance of aINS-NAc circuits in promoting alcohol consumption and the role for aINS OX1Rs in driving nicotine intake. However, OX1R inhibition within the mPFC did reduce alcohol drinking, indicating cortical OXR involvement in promoting intake. Also, in support of the critical role for mNAsh OX1Rs, SB within the mNAsh also significantly reduced operant alcohol self-administration in rats. Finally, orexin *ex vivo* enhanced firing in mNAsh neurons from alcohol-drinking mice, with no effect on evoked EPSCs or input resistance; a similar orexin increase in firing without a change in input resistance was observed in alcohol-naïve mice. Taken together, our results suggest that OX1Rs within the mNAsh and mPFC, but not the aINS, play a central role in driving excessive alcohol drinking.

## Introduction

Addiction to abused substances, including alcohol, is characterized by strong motivation for the addictive substance (Larimer et al., 1999; Sinha, 2009; Koob and Volkow, 2010). However, despite extensive efforts, alcohol use disorders (AUDs) remain a significant problem with substantial personal, social, and economic costs (Harwood et al., 1998; Blincoe et al., 2002; Mokdad et al., 2004; Dawson et al., 2005; Hingson et al., 2005; Rehm et al., 2009; Bouchery et al., 2011; Sacks et al., 2013; CDC, 2014; SAMHSA, 2014), especially because of the limited pharmacotherapies that are effective against AUDs (Spanagel, 2009; WHO, 2014).

Orexin receptors (OXRs) are of particular interest for addictive behaviors since they can promote intake of a number of motivating and addictive substances (Mahler et al., 2012, 2014; Boutrel et al., 2013; Barson and Leibowitz, 2016). OXRs are activated by the neuropeptide orexin, which is synthesized in a subset of cells in the lateral hypothalamus that project throughout the brain (de Lecea et al., 1998), and mediate a variety of regulatory and homeostatic behaviors ranging from sleep-wake cycle and neuroendocrine regulation to feeding and arousal (Mahler et al., 2014; Brown J. A. et al., 2015; Li et al., 2016). Orexin can act through OX1-type or OX2-type receptors (OX1Rs or OX2Rs), and although both receptors can contribute to addictive behaviors (Mahler et al., 2012), existing studies suggest that OX1Rs play a much more important overall role relative to OX2Rs (Moorman and Aston-Jones, 2009; Baimel et al., 2014; Barson et al., 2014; Mahler et al., 2014; Brown J. A. et al., 2015; but see Brown et al., 2013; Anderson et al., 2014). In particular, OX1Rs have been implicated in driving the pursuit and intake of high-value, salient natural rewards, such as sucrose and high-fat foods, as well as addictive substances such as cocaine, opioids, nicotine, and alcohol, with little role in sustaining consumption of less motivating substances (Borgland et al., 2009; Cason et al., 2010; Baimel et al., 2014; Mahler et al., 2014). For example, OX1Rs mediate greater alcohol preference and intake in rats (Moorman and Aston-Jones, 2009) and increased alcohol drinking in dependent mice (Lopez et al., 2016). Thus, OX1R signaling could represent an important and novel clinical and therapeutic target for intervention for AUDs (Khoo and Brown, 2014; Li et al., 2016).

While considerable evidence implicates OX1Rs in driving intake of preferred rewards, the brain regions that mediate this effect on alcohol drinking are poorly understood. We recently demonstrated that projections from the anterior insula (aINS) to the nucleus accumbens (NAc) are critical for driving compulsion-like alcohol drinking in rats (Seif et al., 2013). The medial Shell subregion of the NAc (mNAsh) also plays an important role in a promoting a number of addictive and other motivated behaviors (Anderson et al., 2008; Chaudhri et al., 2010; Saddoris et al., 2013; Castro et al., 2015; Corbit and Balleine, 2015; Marchant et al., 2015; Millan et al., 2015), including a role for mNAsh OXRs during feeding and morphine-related behavior (Thorpe and Kotz, 2005; Qi et al., 2013; Sadeghzadeh et al., 2016), although there have been mixed results regarding the presence of OX1Rs within the mNAsh (See Section Discussion). The aINS is also thought to play a central role in driving addictive behaviors in both humans (Naqvi et al., 2014) and animals (Hollander et al., 2008; Seif et al., 2013), and OX1Rs within the aINS promote nicotine intake (Hollander et al., 2008). In addition, OX1Rs in the mPFC have been shown to increase alcohol relapse (Brown R. M. et al., 2015). Thus, we examined whether OX1Rs in the mNAsh, aINS, and mPFC were important for driving excessive alcohol drinking in mice, and whether mNAsh OX1Rs promoted responding for alcohol in rats. We also used electrophysiology to assess whether OX1Rs altered measures of mNAsh activity *ex vivo*.

## Methods

### Animals

All procedures followed the Guide for Care and Use of Laboratory Animals provided by the National Institutes of Health, and with approval of the Institutional Animal Care and Use Committee of UCSF. Male C57BL/6 mice, 7–8-week of age, were purchased from Jackson Laboratories. Mice were single-housed under a reverse 12:12 light:dark cycle, with lights off at 10:00 a.m. Male Long-Evans rats, 45–50 days of age, were purchased from Harlan and singly housed, and housed under a standard light-dark cycle (with drinking experiments performed in the light cycle). Food and water were available, *ad libitum*, for all subjects. We used mice because of their higher level of drinking under two-bottle intake relative to rats. In contrast, operant methods in mice are much more challenging, and thus we utilized operant methods in rats.

### Limited daily access (LDA) to alcohol in mice

The repeated, limited access choice alcohol drinking model was modified from that previously described (Lesscher et al., 2010; Kasten and Boehm, 2014). Mice were first acclimated to housing conditions for 2-week. Mice were then given two-bottle choice access to one bottle with 15% alcohol (v/v) in water and a second bottle of water. Mice first had a 24-h overnight alcohol-drinking session, followed by a 24-h withdrawal period. Thereafter, mice were presented daily with 15% alcohol and water for 2-h, Monday–Friday, in their home cage, with drinking sessions starting 3-h into the dark cycle. This excessive-drinking exposure paradigm leads to binge levels of alcohol drinking (>80 mg%) (Lei et al., 2016). In order to control for side preference, the bottle placements of the solutions were alternated between each drinking session. Intake level was measured by determining bottle weight and corrected for spill, which was determined by separate spill-control bottles.

### Saccharin intake in mice

**Figure 1:**
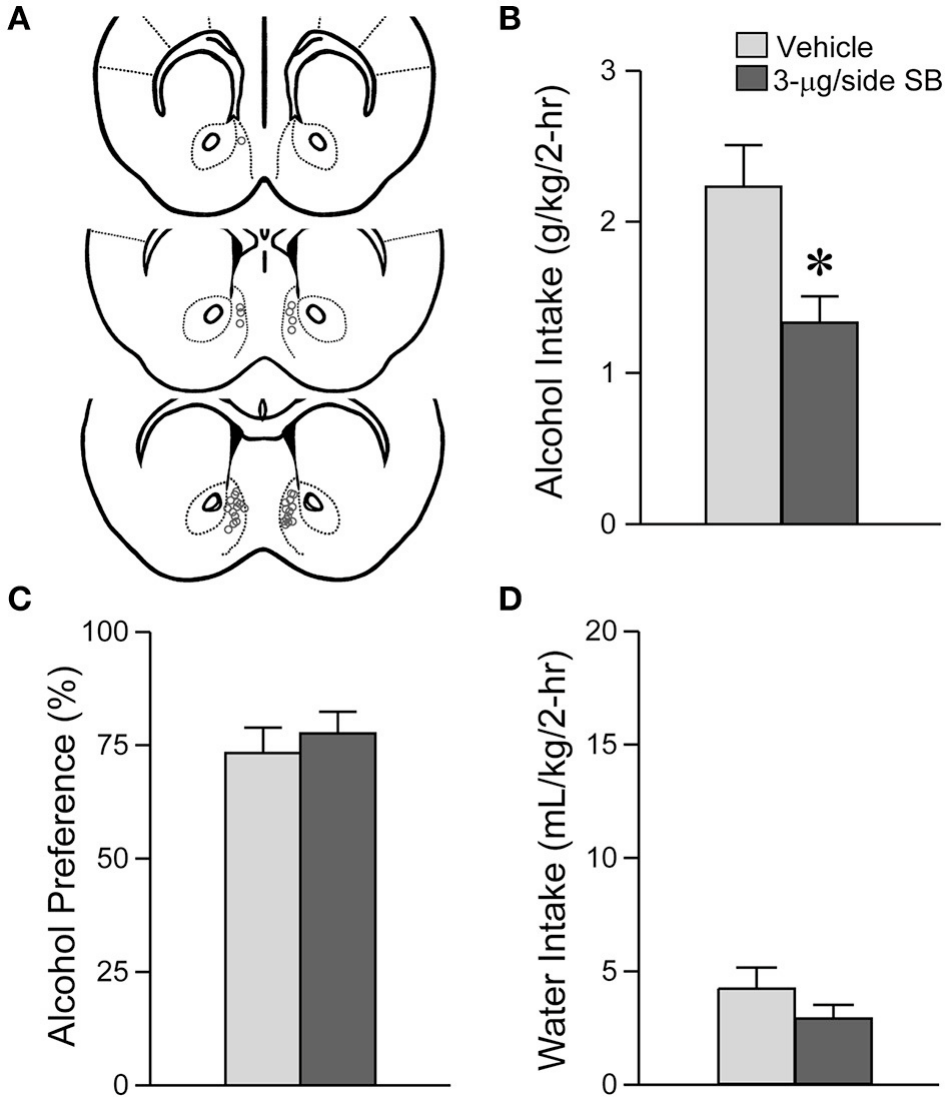
**OX1R blockade within the medial NAc Shell significantly reduced alcohol drinking in mice**. **(A)** Locations of cannulae shown by gray circles; sections at approximately AP +1.34, +1.18 and +0.98 mm relative to Bregma. **(B)** Infusion of 3-μg SB within the mNAsh decreased alcohol intake. **(C,D)** No changes in **(C)** alcohol preference or **(D)** concurrent water intake. Preference was calculated as (ml alcohol)/(ml alcohol + ml water). ^*^p < 0.05.

**Figure 2:**
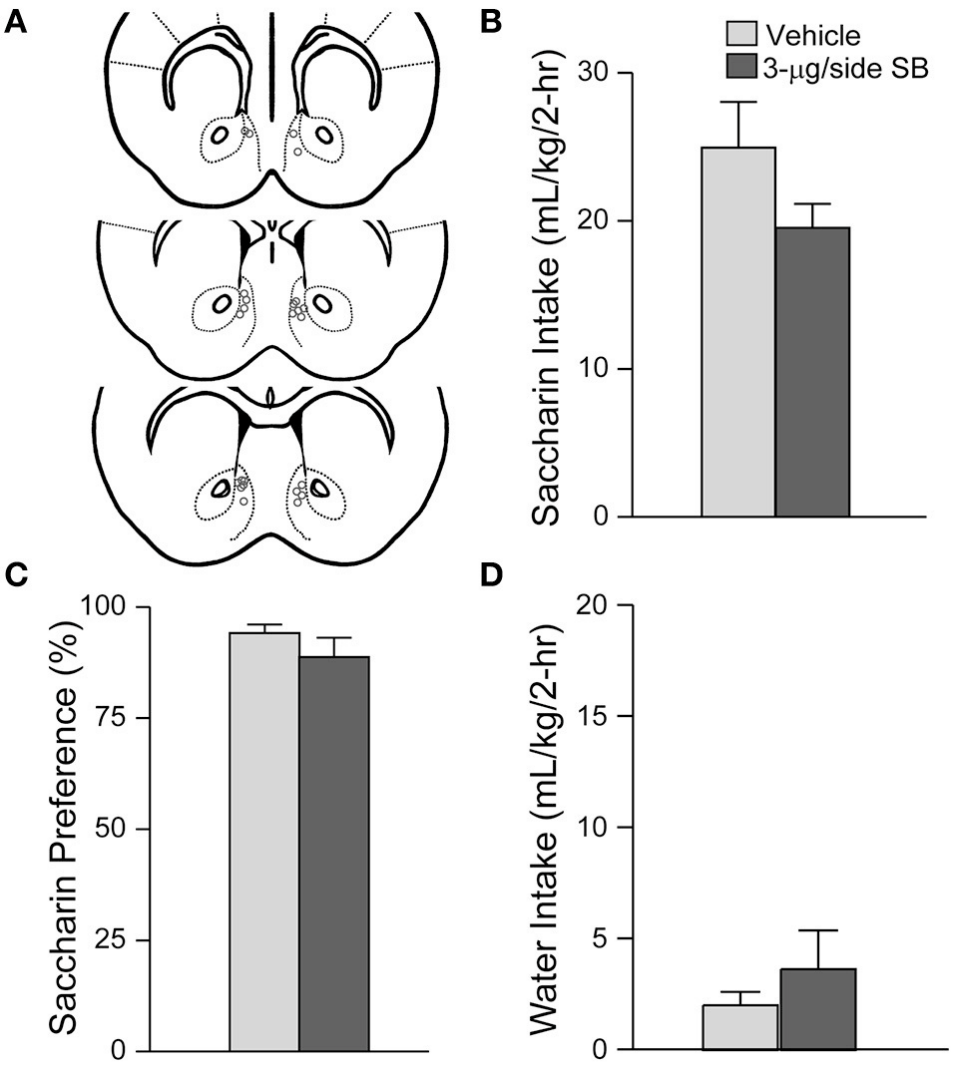
**mNAsh OX1R blockade did not alter saccharin drinking**. **(A)** Locations of cannulae, as for Figure 1. **(B–D)** Infusion of 3-μg SB within the mNAsh did not alter **(B)** saccharin intake, **(C)** preference or **(D)** concurrent water intake.

Drinking of a 0.05% saccharin solution under two-bottle choice was performed using a schedule identical to that used for alcohol. This concentration was determined to give approximately the same volume of intake as alcohol (e.g., 19.2 ± 2.3 ml/kg/2-h of alcohol intake for vehicle condition in Figure 1B; *t*_(1, 27)_ = 1.54, *p* = 0.14 vs. volume of saccharin intake for vehicle condition in Figure 2B).

### Alcohol self-administration in rats

Rat self-administration methods were identical to those previously described (Simms et al., 2011b), using standard operant conditioning chambers (MedAssociates). Briefly, rats underwent six 14-h overnight session on an FR1 schedule, with 0.1 ml of 20% alcohol delivered after each FR1 press. During overnight training, only the active lever was available, which allowed the establishment of lever-pressing behavior. Rats then had six sessions of 45-min FR1, and then were moved to a daily 30-min FR3 schedule of responding; a second, inactive lever was also introduced during the FR3 sessions. In all phases of training, successful completion of an FR response resulted in alcohol delivery as well as a cue light above the active lever and a tone. Inactive lever presses were not accompanied by alcohol delivery or light or tone cues. Rats pressed for a minimum of 20 sessions before testing the impact of SB on responding for alcohol. Any animal receiving less 0.3 g/kg ethanol intake per session were excluded (one rat in this study).

### Cannula implantation surgeries

In mice, after ~2-week of LDA, surgery was performed to implant bilateral guide cannulae (Plastics One) aimed at the mNAsh (AP +1.5, ML ±0.5, DV −4.5 mm), an offsite control region above the mNAsh (AP +1.5, ML ±0.5, DV −3.0 mm), aINS (AP +2.0, ML ±2.4, DV −2.0 mm), and the mPFC (attempting to target the infralimbic) (AP +1.7, ML ±0.4, DV −2.7 mm). In rats, after establishing FR3 responding, bilateral guide cannulae were implanted targeting the mNAsh (AP +1.8, ML ±0.8, DV −6.5 mm). All coordinates are given relative to Bregma. After surgery, animals were allowed to recover for 1-week before resuming alcohol drinking sessions, and handling for drug microinfusions began the week after. After completion of drug treatments, brains were harvested for verification of cannula placement.

### Drug microinfusions

SB-334867 (SB, Tocris), a selective OX1R antagonist, was dissolved in 100% DMSO vehicle (Simms et al., 2011a). Mice received microinjections of either vehicle or 3-μg/200 nL/side (47 nmol) of SB 30-min prior to an alcohol-drinking session, or 3-μg/200 nL/side (59 nmol) of the OX2R antagonist TCS-OX2-29 (TCS, Tocris); these are relatively high doses but have previously been utilized (Borgland et al., 2006; Qi et al., 2013; Brown R. M. et al., 2015). Drugs were injected with bilateral infusion needles (Plastics One) that projected 0.3 mm past the end of the guide cannulae, at a rate of 200 nL/min. Needles were left in place for an extra 1-min before retraction. Each dose (vehicle and 3-μg SB or TCS) was microinjected twice (on different test days) and counter-balanced across treatment and animals.

Rats received microinjections of either 0- or 3-μg/600 nL/side of SB, on different days using a within-animal design in a counter-balance manner, 30-min prior to alcohol self-administration sessions. Drugs were injected 600 nL/min via bilateral infusions needles (Plastics One) that project 1-mm beyond the guide cannulae. Needles were left in place for an extra 90-s.

Mice or rats were given at least 1 day of alcohol or saccharin drinking between test days.

Alcohol intake, especially bottle drinking, can be influenced by handling (Lum et al., 2014), and thus it is important to give animals a period of time after injection to remove possible confounds of handling on alcohol drinking, and we used a 30 min time point between drug injection and behavioral testing. Thus, given the use of DMSO as a vehicle for SB, we cannot completely rule out the possibility that this period of time might have allowed greater diffusion of OXRS to adjacent brain regions, for example into the adjacent NAcore. However, even if a briefer time was used between injection and initiation of alcohol drinking, the drinking sessions were 2 h and any such issues related to diffusion would become apparent within the first hour of drinking. Also, although we do not have specific information about the level of spread of our infusate, 200 nl has previously used as an microinjection volume for studies distinguishing mNAsh vs. NAcb Core in mice (Managò et al., 2008), and is widely used as a volume for injection for other studies in mice (e.g., Stratford and Wirtshafter, 2011; Kasten and Boehm, 2014; Ramaker et al., 2015).

DMSO as vehicle could have effects on drinking. However, alcohol intake in the presence of intra-mNAsh DMSO infusion was not different from alcohol drinking levels in the uninjected days adjacent to days with DMSO infusion [DMSO infusion: 2.23 ± 0.28 g/kg; adjacent uninjected days: 2.31 ± 0.23 g/kg; *t*_(1, 16)_ = 0.26, *p* = 0.80; *n* = 17, determined for the mice shown in Figure 1, DMSO results are same as shown in Figure 1]. Also, other studies have demonstrated that intracranial injection of DMSO does not have non-specific effects on behavior relative to saline injection (e.g., Naghdi and Asadollahi, 2004). Thus, the DMSO vehicle itself likely did not have non-specific effects on alcohol drinking.

### Brain slice preparation and *ex vivo* electrophysiology

Slice preparation and electrophysiology methods were similar to those previously described (Seif et al., 2011, 2013; Pomrenze et al., 2015). Briefly, adult mice were anesthetized with pentobarbital (100 mg/kg), decapitated, and brain slices were cut in an ice-cold glycerol-based solution (in mM: 252 glycerol, 2.5 KCl, 1.25 NaH_2_PO_4_, 1 MgCl_2_, 2 CaCl_2_, 25 NaHCO_3_, 1 L-ascorbate, and 11 glucose, bubbled with carbogen) (Pomrenze et al., 2015). Slices recovered at 32°C in carbogen-bubbled aCSF (containing, in mM: 126 NaCl, 2.5 KCl, 1.2 NaH_2_PO_4_, 1.2 MgCl_2_, 2.4 CaCl_2_, 18 NaHCO_3_, 11 glucose, pH 7.2–7.4, mOsm 302–305) for at least 30 min before experiments, with 1 mM ascorbic acid added just before the first slice. During experiments, slices were submerged and perfused (2 ml/min) with aCSF, 31–32°C, with CNQX (10 μM) and picrotoxin (50 μM). Action potential firing and EPSCs were recorded using Clampex 10.1 and an Axon 700 A patch amplifier (Axon Instruments, Foster City, CA). All experiments were performed using whole-cell recording using visualized infrared-DIC with 2.5–3.5 M electrodes.

Firing and input resistance were measured using a potassium-methanesulfonate based internal solution (in mM: 130 KOH, 105 methanesulfonic acid, 17 HCl, 20 HEPES, 0.2 EGTA, 2.8 NaCl, 2.5 mg/ml Mg-ATP, 0.25 mg/ml GTP, pH 7.2–7.4, 278–287 mOsm). DC current was passed to bring each neuron to ~-85 mV before starting firing experiments. Rheobase (minimum current needed to generate firing) was first identified for each cell by a series of 500 ms current steps, increasing in 20 pA increments, which was then terminated once rheobase was identified. We then began experiments where a more restricted set of 500 ms current steps (6–7 steps, 20 pA apart, with the first pulse subthreshold for firing) which were repeated every 30 s to measure possible changes in firing across the duration of the experiment. Depolarizing pulses alternated with a 30 pA hyperpolarizing pulse to examine the input resistance. Changes in firing and input resistance with 10 min of orexinA application (100 nM) was determined after ~15 min baseline, with SB added 5 min before orexinA exposure in some cells.

To determine orexinA-related changes in firing, we averaged 7 min of baseline, and averaged the last 7 min of the 10-min orexinA exposure (since it usually takes 2–3 min before a drug effect is clear); we then subtracted the average number of action potentials generated during orexinA exposure from average number of spikes at baseline. This was determined at rheobase, the minimum current where firing was evoked, and at the current step above rheobase. Rheobase was 168 ± 24 pA (range: 100–320 pA) for experiments from alcohol-drinking mice, and 185 ± 22 pA (range: 125–240 pA) for alcohol-naïve experiments.

EPSCs were measured using a cesium-methanesulfonate based internal solution (in mM: 120 cesium methanesulfonate, 20 HEPES, 0.4 EGTA, 2.8 NaCl, 5 TEA chloride, 2.5 Mg-ATP, 0.25 Na-GTP, pH 7.2–7.3, 270–285 mOsm). EPSCs were filtered at 2 kHz and digitized at 10 kHz. Series resistance (10-30 MΩ) and input resistance were monitored on-line using a 4-mV depolarizing step (50 ms) which was given after every EPSC. Electrically-evoked currents were elicited using a bipolar stimulating electrode placed ~200 μM dorsal to the recording site. As with firing, 7 min of baseline and orexinA exposure were used for determining the percent change in EPSCs with orexinA.

Statistics for electrophysiology experiments were performed either using an unpaired *t*-test, to compare changes in firing in orexin-exposed neurons with or without SB pre-exposure, or using a paired *t*-test, to examine whether orexin exposure altered input resistance or EPSCs relative to pre-orexin baseline under a particular condition.

### Data analyses for behavioral studies

In mice, after each drinking session, the water and alcohol (or saccharin) bottles were weighed; subsequently, these values were used to determine the intake of alcohol (g/kg of body weight) or saccharin (mL/kg of body weight), as well as water (ml/kg of body weight) and the preference ratio for the alcohol or saccharin (volume of reward intake/total volume of reward plus water intake). Due to variability of two-bottle choice alcohol drinking in mice, each mouse had two test sessions for vehicle and two test sessions for OXR blocker, and the average of the two drinking sessions for each treatment was used for a given animal. Importantly, all such tests were performed in a counterbalanced order: a mouse received vehicle vs. drug in a counterbalanced order for the first two test sessions, such that half the animals received vehicle in the first session, and the other half received OXR blocker in the first session. The same counterbalanced order was then used for the third and fourth test sessions, to insure that animals did not receive the same treatment in consecutive sessions, and to minimize the order effects. Rats received one test session of vehicle or SB, which was counterbalanced across animals, and active and inactive lever pressing, rewards received, and g/kg intake levels, were all determined. Finally, since the effects of vehicle and drug were tested within the same animal, all statistics for behavioral experiments were performed using a paired *t*-test, using SPSS (IBM). Different behavioral measures were assessed by separate paired *t*-tests. All data are shown as mean ± SEM.

In our experience, alcohol intake levels in daily two-bottle choice sessions are more variable relative to operant responding, and bottle drinking in mice is more variable than in rat. Thus, we have adopted the method in mice where vehicle is tested twice and the two sessions averaged, and a given agent is tested twice then averaged. In addition, to determine test-retest variance, we examined the data for intra-mNAsh vehicle vs. SB during alcohol drinking. We first calculated the difference between the second-vehicle and first-vehicle test sessions that an animal underwent, or between the second-drug and first-drug test sessions. The standard deviation of test-retest was 2.54 g/kg for vehicle and 1.35 g/kg for SB. Although the test-retest variability was larger for vehicle, animals drank an average of 2.23 g/kg alcohol for vehicle sessions and 1.33 g/kg alcohol for SB sessions. Thus, the somewhat larger test-retest variability for vehicle sessions may reflect the larger volume of intake during vehicle sessions. In addition, to assess order effects, we performed a two-way RM ANOVA with drug vs. vehicle as one factor and the first vs. second test session of the given agent as a second factor. There were no significant effects of first vs. second session [*F*_(1, 32)_ = 1.647, *p* = 0.209], or interaction of session number with group [vehicle or drug; *F*_(1, 32)_ = 0.009, *p* = 0.924], although there was a significant effect of group [*F*_(1, 32)_ = 7.602, *p* = 0.010], consistent with our previous analysis that SB in the mNAsh reduced alcohol drinking relative to vehicle. These findings suggest that there were no differences in intake between the first and second test sessions for either vehicle or drug.

One limitation of the present experiments is the possibility that some experiments may be underpowered to observe a change in intake with OXR blockers, e.g., the trend for intra-mNAsh SB to decrease in intake in saccharin-consuming mice with *n* = 12 vs. *n* = 17 for alcohol-drinking mice. However, we were able to observe a significant depression of alcohol drinking in mice where SB was injected into the mPFC, with *n* = 9, suggesting that saccharin-intake experiments were sufficiently powered to detect any OXR-relate changes. For the off-site control experiments, it is clear even with *n* = 6 that there is no impact of SB on alcohol drinking, and if anything there is a trend for an increase in preference for alcohol (rather than inhibition of alcohol-related behavior). Nonetheless, with the smaller sample size, the off-site control group could be underpowered to detect differences in alcohol drinking.

## Result

### OX1R blockade in the medial NAc shell suppressed excessive alcohol drinking in mice

We first examined whether OX1Rs in the mNAsh could regulate voluntary excessive alcohol drinking. In particular, we microinjected vehicle or a previously used concentration of the OX1R-selective antagonist, SB-334867 (SB, 3-μg/side) (Hollander et al., 2008; Espana et al., 2010; Plaza-Zabala et al., 2012), prior to an LDA drinking session using a counter-balanced, within-animal design (*n* = 17). Our results showed that inhibition of OX1Rs within the mNAsh (Figure 1A) significantly decreased alcohol drinking [Figure 1B; *t*_(1, 16)_ = 2.78, *p* = 0.013]. No changes were observed in preference [Figure 1C; *t*_(1, 16)_ = 0.58, *p* = 0.57], which likely reflects the low volume of concurrent water intake during the 2-h alcohol-drinking sessions [Figure 1D; *t*_(1, 16)_ = 1.50, *p* = 0.15; see also Dhaher et al., 2009; Seif et al., 2015; den Hartog et al., 2016]. Thus, our results suggest that OX1Rs within the mNAsh are important for driving alcohol drinking.

Suppression of alcohol drinking by the OX1R antagonist in the mNAsh might represent non-specific changes in motor function or consumption. Thus, we next examined whether SB within the mNAsh (Figure 2A) would reduce intake of 0.05% saccharin (*n* = 12). However, 3-μg of SB within the mNAsh had no effect on saccharin consumption [Figure 2B; *t*_(1, 11)_ = 1.68, *p* = 0.12], preference [Figure 2C; *t*_(1, 11)_ = 0.97, *p* = 0.35], or concurrent water intake [Figure 2D; *t*_(1, 11)_ = 0.77, *p* = 0.46]. Thus, the suppression of alcohol drinking when infusing OX1R inhibitors within the mNAsh was likely due to a particular role in promoting alcohol drinking, rather than through more general regulation of consumption or activity. However, we do note that there was a trend for a decrease in saccharin intake with intra-mNAsh SB infusion.

**Figure 3:**
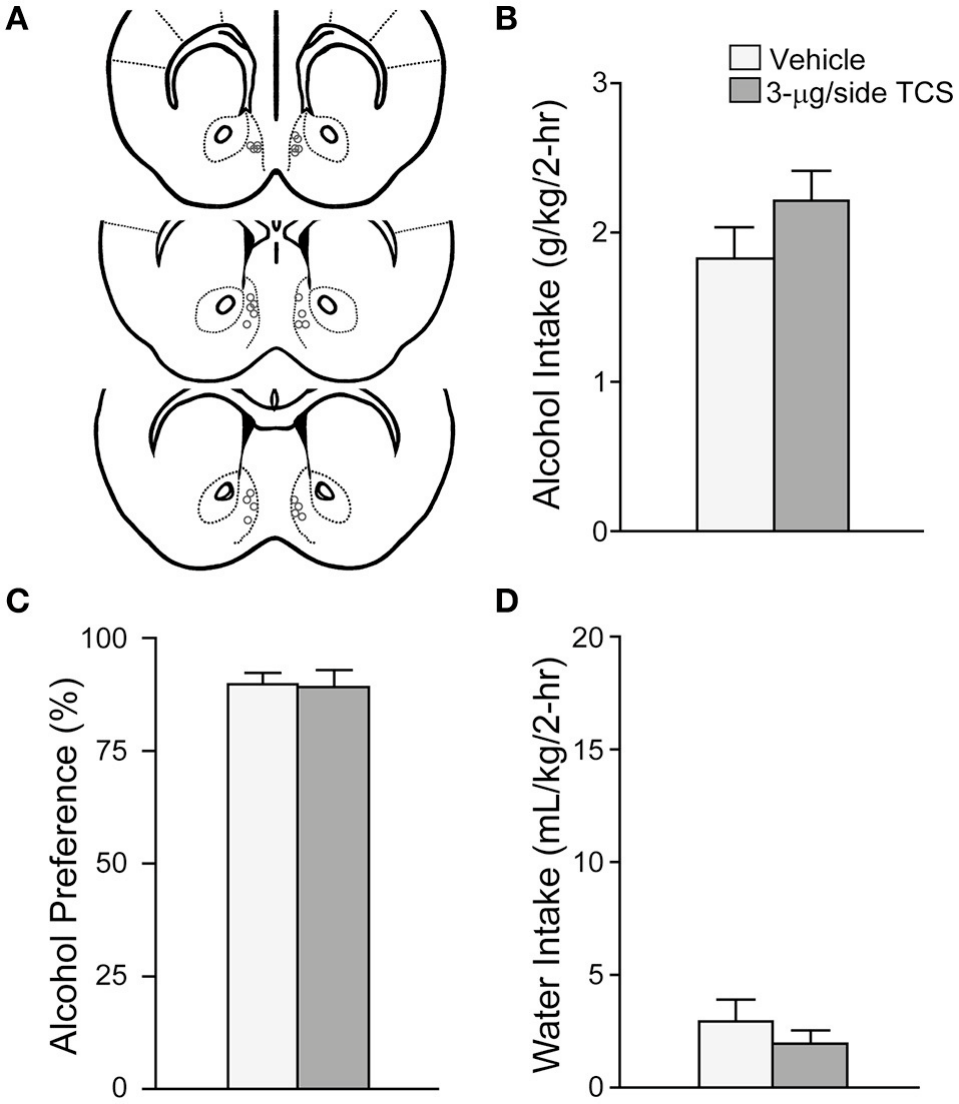
**OX2R blockade within the mNAsh did not reduce alcohol drinking**. **(A)** Locations of cannulae, as for Figure 1. **(B–D)** Infusion of 3-μg TCS within the mNAsh did not alter **(B)** alcohol intake, **(C)** preference, or **(D)** concurrent water intake.

To assess the possible involvement of OX2Rs in alcohol drinking, we tested whether injection of the OX2R blocker TCS-OX2-29 (3-μg) into the mNAsh (Figure 3A; *n* = 13) would alter excessive alcohol consumption, similar to what we observed with intra-mNAsh SB. However, TCS within the mNAsh did not reduce alcohol intake [Figure 3B; *t*_(1, 12)_ = 1.94, *p* = 0.08], and if anything had a trend to increase intake. TCS within the mNAsh also did not alter preference [Figure 3C; *t*_(1, 12)_ = 0.14, *p* = 0.89] or concurrent water intake [Figure 3D; *t*_(1, 12)_ = 1.27, *p* = 0.23]. These results indicate that OX1Rs but not OX2Rs within the mNAsh were important for promoting excessive alcohol drinking in mice.

**Figure 4:**
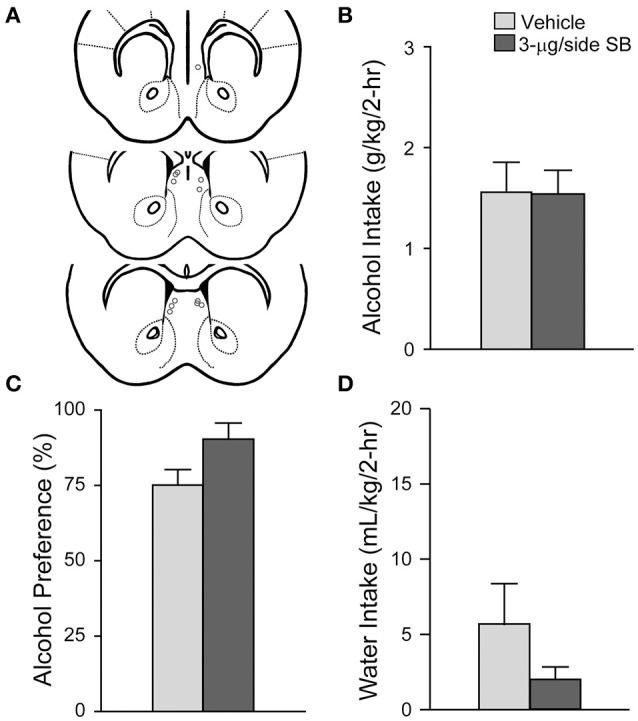
**OX1R blockade at an off-site control above the mNAsh did not alter alcohol drinking**. **(A)** Locations of cannulae, as for Figure 1. **(B–D)** Infusion of 3-μg SB ~1.5 mM above the mNAsh did not alter **(B)** alcohol intake, **(C)** preference, or **(D)** concurrent water intake.

To test whether SB could affect alcohol drinking by diffusing and acting in a region adjacent to the mNAsh, we administered the same dose and vehicle into an off-site control region 1.5-mm dorsal to the mNAsh (*n* = 6; Figure 4A). However, no changes in alcohol intake were observed when 3-μg SB was injected into the off-site region control group [Figure 4B; *t*_(1, 5)_ = 0.04, *p* = 0.97], and with no change in alcohol preference [Figure 4C; *t*_(1, 5)_ = 2.22, *p* = 0.077] or concurrent water intake [Figure 4D; *t*_(1, 5)_ = 1.56, *p* = 0.18]. Thus, the apparent mNAsh OX1R promotion of alcohol drinking was unlikely to reflect action of SB in brain areas above the mNAsh.

**Figure 5:**
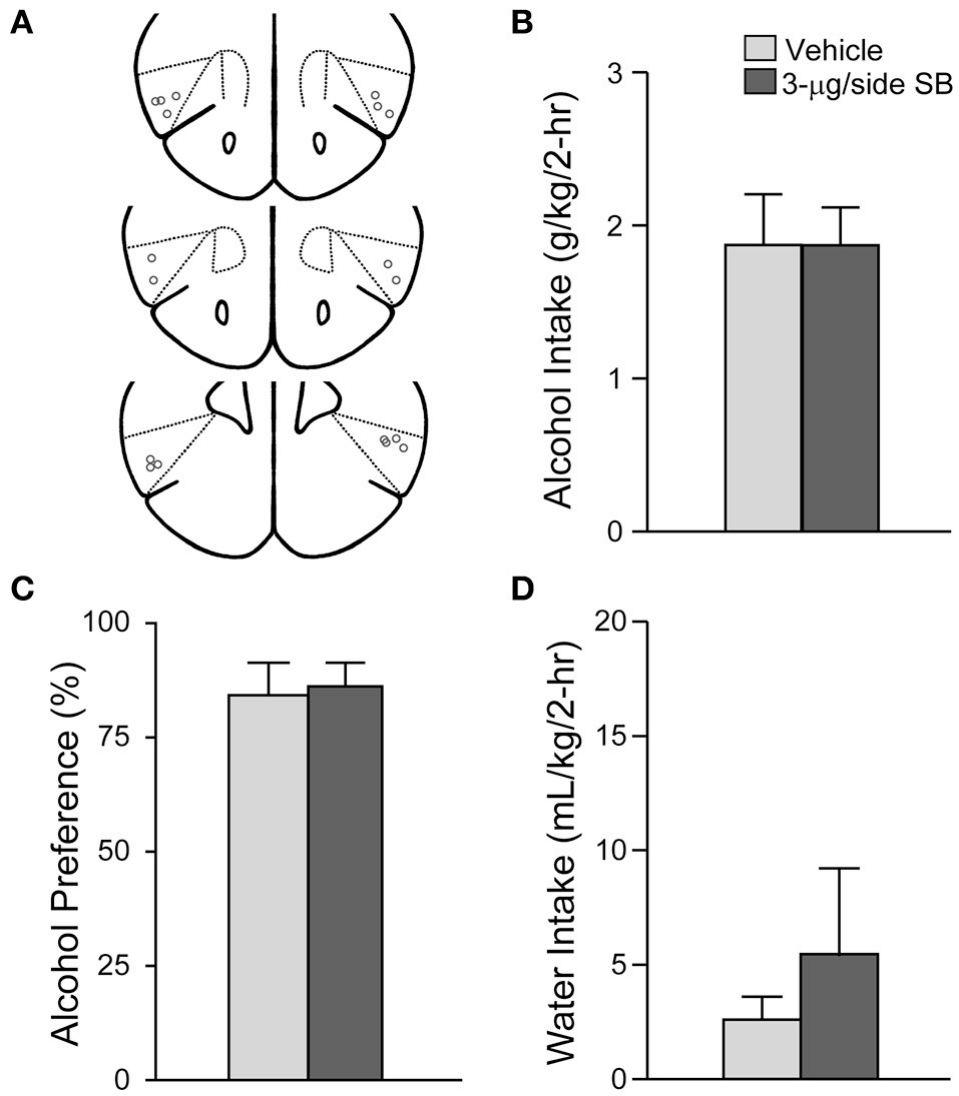
**OX1R blockade in the anterior Insular cortex did not alter alcohol drinking**. **(A)** Locations of cannulae shown by gray circles; sections at approximately AP +2.22, +2.10, and +1.98 mm relative to Bregma. **(B–D)** Infusion of 3-μg SB within the aINS did not alter **(B)** alcohol intake, **(C)** preference, or **(D)** concurrent water intake.

In addition to the mNAsh, the aINS likely plays a central role in promoting many addiction-related behaviors. Thus, we also examined whether OX1Rs within the aINS would promote alcohol drinking (*n* = 9) (Figure 5A), as was observed for OX1Rs within the mNAsh (Figure 1). However, infusion of SB within the aINS had no effect on alcohol intake [Figure 5B; *t*_(1, 8)_ = 0.03, *p* = 0.98], preference [Figure 5C; *t*_(1, 8)_ = 0.17, *p* = 0.87] or concurrent water intake [Figure 5D; *t*_(1, 8)_ = 0.73, *p* = 0.49]. Thus, contrary to our predictions, OX1Rs within the aINS did not play a role in promoting alcohol drinking in mice.

**Figure 6:**
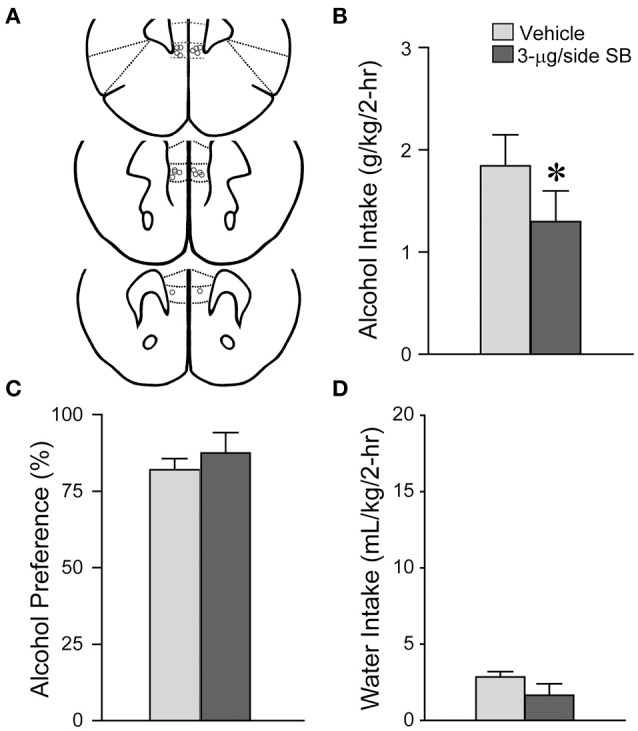
**OX1R blockade in the mPFC significantly reduced alcohol drinking**. **(A)** Locations of cannulae shown by gray circles; sections at approximately AP +1.98, +1.78, and +1.70 mm relative to Bregma. **(B)** Infusion of 3-μg SB within the mPFC decreased alcohol intake. **(C,D)** No changes in **(C)** alcohol preference or **(D)** concurrent water intake. ^*^p < 0.05.

Finally, other cortical areas have also been implicated in regulating alcohol drinking, including the medial prefrontal cortex. In agreement, we found that infusion of 3-μg SB within the mPFC (Figure 6A; *n* = 9) significantly reduced alcohol drinking [Figure 6B; *t*_(1, 8)_ = 2.34, *p* = 0.048], with no impact on preference [Figure 6C; *t*_(1, 8)_ = 0.75, *p* = 0.47] or concurrent water intake [Figure 6D; *t*_(1, 8)_ = 1.69, *p* = 0.13]. Thus, our results together suggest that mPFC but not aINS OX1Rs are important for promoting alcohol drinking.

### NAsh OX1R blockade decreased lever pressing for alcohol in rats

**Figure 7:**
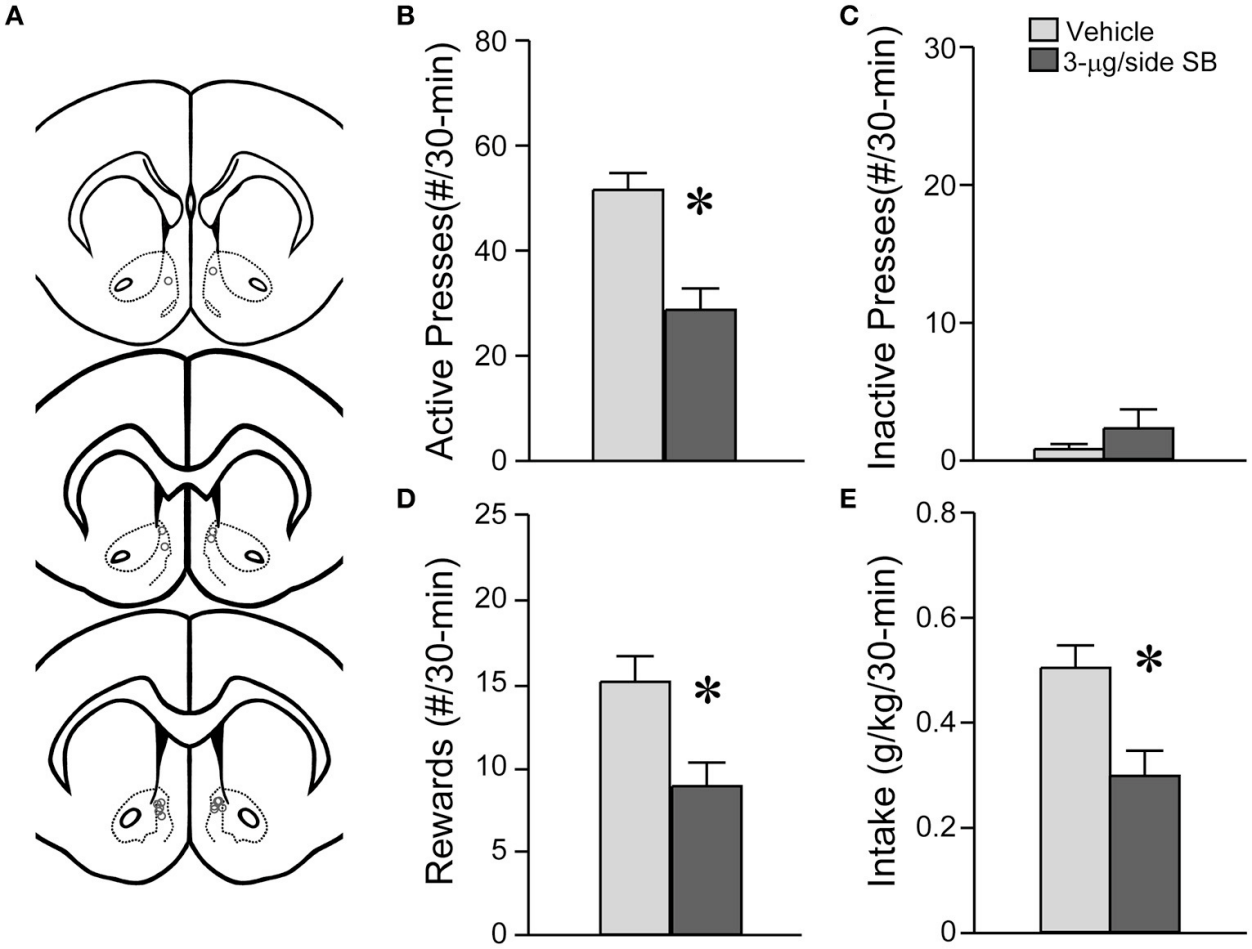
**OX1R blockade within the medial NAc Shell significantly reduced operant alcohol drinking in rats**. **(A)** Locations of cannulae shown by gray circles; sections at approximately AP +1.70, +1.60, and +1.20 relative to Bregma. **(B)** Infusion of 3-μg SB within the mNAsh of rats decreased active lever presses for alcohol. **(C)** No changes in inactive lever presses with SB. **(D,E)** OX1R inhibition in the mNAsh reduced **(D)** rewards received and **(E)** alcohol intake levels. ^*^p < 0.05.

Since OX1R inhibition within the mNAsh of mice significantly reduced alcohol drinking, we next examined whether mNAsh OX1Rs would be important for promoting alcohol intake under a different drinking model, operant responding for alcohol in rats (*n* = 8) (Figure 7A). In fact, inhibition of OX1Rs within the mNAsh of rats with 3-μg SB significantly reduced lever-pressing for alcohol, tested within-rat vs. vehicle [Figure 7B; *t*_(1, 7)_ = 4.17, *p* = 0.004], with an ~40% reduction, similar to what was observed in mice. OX1R inhibition had no effect on pressing of the inactive lever [Figure 7C; *t*_(1, 7)_ = 1.34, *p* = 0.22], although this was already very low. In addition, OX1R inhibition within the mNAsh significantly reduced the number of rewards received [Figure 7D; *t*_(1, 7)_ = 2.95, *p* = 0.021] and the amount of alcohol consumed [Figure 7E; *t*_(1, 7)_ = 2.84, *p* = 0.025]. Thus, OX1Rs in the mNAsh were critical for promoting alcohol consumption in both mice and rats.

### OrexinA peptide enhanced mNAsh action potential firing through OX1Rs

**Figure 8:**
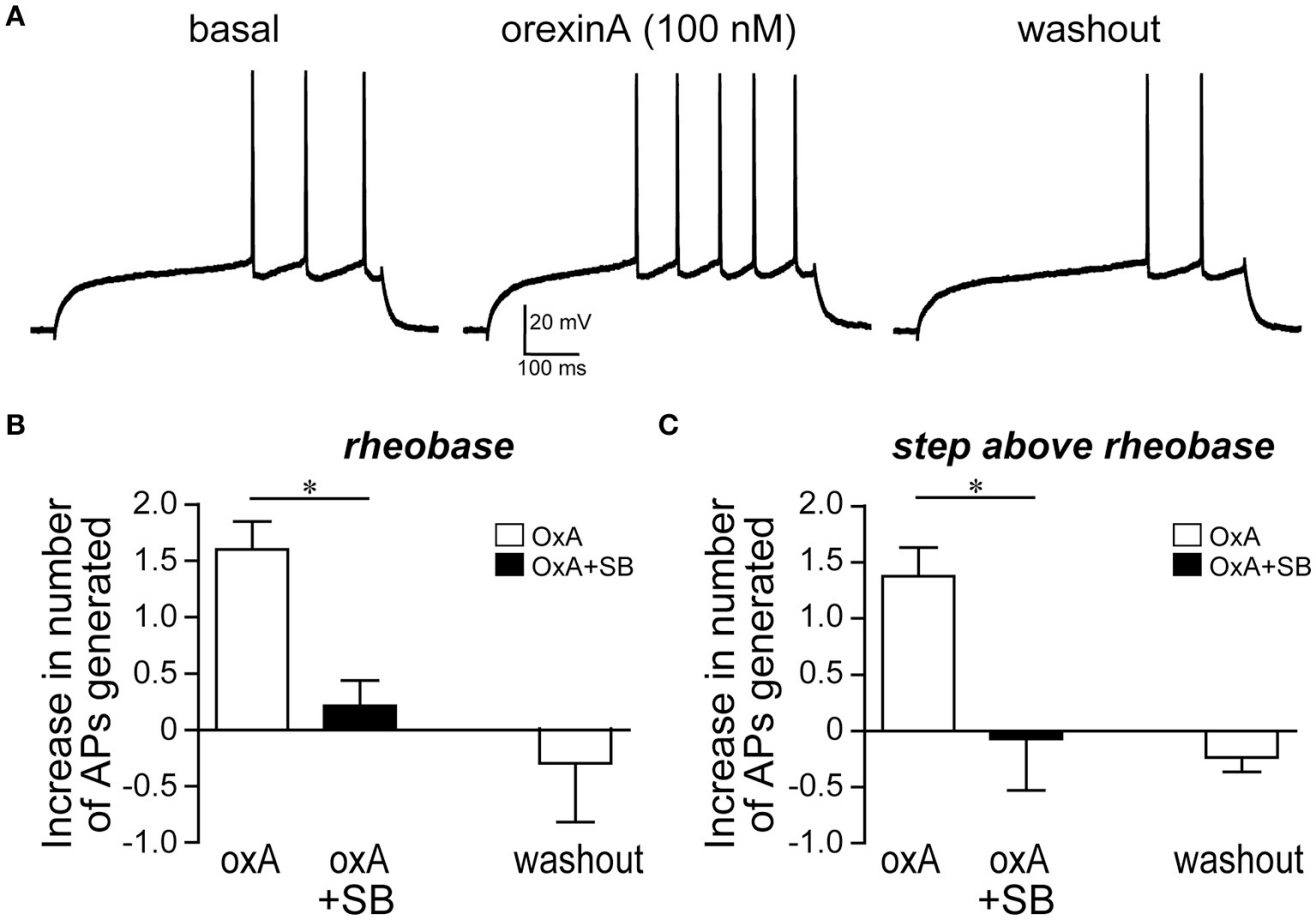
**OrexinA increased firing *ex vivo* in medial NAc Shell neurons from alcohol-drinking mice. (A)** Example showing that 100 nM orexinA (OxA) increased firing in the mNAsh neurons from adult alcohol-drinking mice; firing was evoked by 500-ms depolarizing current pulses (See Section Methods). Example is from rheobase, the minimum current required to evoke firing, in this neuron **(B,C)** Averaged data showing the increased number of action potentials generated with OxA **(B)** at rheobase, and **(C)** at the current step above rheobase, and that blocking OX1Rs with SB prevented the OxA increase in firing. ^*^p < 0.05.

**Figure 9:**
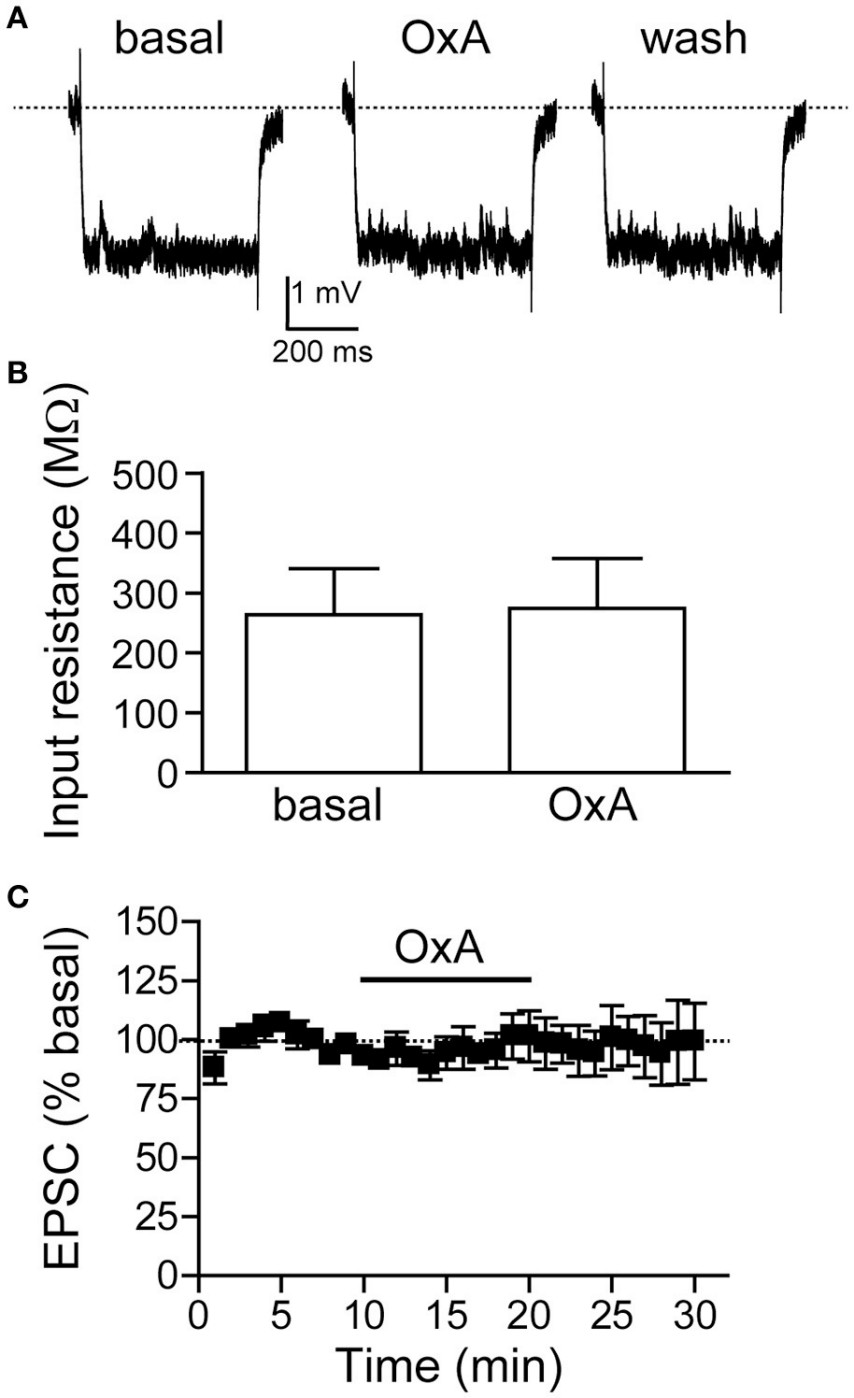
**OrexinA *ex vivo* did not alter input resistance or evoked EPSCs in medial NAc Shell neurons from alcohol-drinking mice. (A,B)** Examples **(A)** and grouped data **(B)** showing that OxA did not change input resistance (30 pA hyperpolarizing step). **(C)** Plot across time showing that OxA did not change evoked EPSCs at −70 mV.

Since OX1Rs within the mNAsh were important for promoting alcohol drinking, we next examined whether orexin would impact mNAsh firing *ex vivo* in brain slices from adult alcohol-drinking mice. In fact, firing evoked by depolarizing current pulses was significantly enhanced in the presence of orexinA (100 nM, *n* = 5), which was apparent when analyzing both at rheobase, the minimum current required to evoke action potentials in a cell [Figures 8A,B; paired *t*_(4)_ = 6.54, *p* = 0.003], and at the current step above rheobase [Figure 8C; paired *t*_(4)_ = 5.35, *p* = 0.006]. Importantly, this effect of orexinA was prevented by inhibition of OX1Rs with SB [3 μM, *n* = 4; Figures 8B,C; orexinA application with vs. without SB: *t*_(7)_ = 4.09, *p* = 0.005 at rheobase; *t*_(7)_ = 2.91, *p* = 0.023 at step above rheobase]. The orexinA increase in firing was not accompanied by any changes in input resistance, measured using a hyperpolarizing current pulse at resting potential [Figures 9A,B; 2.3 ± 2.7% change in R-input; paired *t*_(4)_ = 1.26, *p* = 0.28]. In addition, orexinA did not alter EPSCs generated at a −70 mV resting potential [Figure 9C; *n* = 6; −3.7 ± 6.5% change in EPSC; paired *t*_(5)_ = 0.21, *p* = 0.84]; this EPSC predominantly reflects AMPA receptor currents (Seif et al., 2013). Together, these results suggest that OX1Rs enhanced mNAsh activity in alcohol-drinking mice through altering intrinsic excitability but not glutamatergic function at −70 mV.

We also examined whether orexinA would alter firing in mNAsh neurons from alcohol-naïve mice. OrexinA (100 nM) significantly increased action potential firing by 1.6 ± 0.5 spikes at rheobase [paired *t*_(4)_ = 3.30, *p* = 0.03; *n* = 5], which was not different from the orexin change in firing in alcohol-drinking mice [from Figure 8B; unpaired *t*_(8)_ = 0.01, *p* = 0.99]. In addition, in agreement with results from alcohol-drinking mice, the orexin enhancement in firing in alcohol-naïve mice was not accompanied by any changes in input resistance [baseline: 307 ± 49 MΩ; orexin: 320 ± 48 MΩ; 4.7 ± 4.6% change in input resistance; paired *t*_(4)_ = 1.20, *p* = 0.30]. Thus, orexin enhanced action potential firing in mNAsh neurons from both alcohol-naive and alcohol-drinking mice.

## Discussion

Alcohol addiction is a significant economic and social problem, in part due to the strong motivation for alcohol that persons with AUDs exhibit. OX1Rs promote intake when there is strong drive for a reward, but the brain region(s) that mediate the OX1R promotion of excessive alcohol drinking remain largely unknown. Here, we demonstrate that the medial NAc Shell (mNAsh) is a critical region where OX1Rs act to promote excessive alcohol intake in mice. Inhibition of OX1Rs within the mouse mNAsh had no effect on consumption of saccharin or concurrent water drinking during alcohol drinking sessions, suggesting that mNAsh OX1R regulation of excessive alcohol intake was not due to nonspecific effects on motor activity or consumption. In addition, inhibition of OX2Rs within the mNAsh did not alter alcohol intake, suggesting a receptor-selective effect within the mNAsh. In addition, OX1R inhibition at an off-site control region above the mNAsh did not alter alcohol drinking. Surprisingly, OX1R inhibition within the mouse aINS also had no effect on excessive alcohol intake drinking, although aINS-NAc circuits are known to promote alcohol consumption and aINS OX1Rs drives nicotine intake. In contrast, OX1Rs within the mPFC did promote alcohol consumption. Further supporting the central role for mNAsh OX1Rs in promoting alcohol drinking, OX1R inhibition within the mNAsh of rats significantly reduced operant alcohol self-administration. Finally, orexin application *ex vivo* significantly enhanced action potential firing of mNAsh neurons from alcohol-drinking mice, with no changes in input resistance or evoked EPSCs. Together, our results suggest that the mNAsh and mPFC, but not the aINS, are critical regions where OX1R activation drives excessive alcohol drinking.

While a number of studies suggest that OX1Rs play a prominent and selective role in responding for more motivating rewards (See Section Introduction), very little is known about the brain regions where OX1Rs act to promote excessive alcohol drinking. Here, we did not assess motivation directly, but instead examined the impact of orexin on drinking behaviors. Our findings are important since they identify the mNAsh as a region where OX1Rs drive alcohol consumption in both mice and rats. In addition, our studies indicate that OX2Rs within the mNAsh are not required to promote excessive alcohol consumption. Previous work has implicated the mNAsh in different forms of addictive and consummatory behaviors, including feeding (Baldo et al., 2013; Richard et al., 2013) and alcohol drinking (Kasten and Boehm, 2014; Lum et al., 2014; Wilden et al., 2014; Ramaker et al., 2015). The mNAsh also promotes different forms of reinstatement (Anderson et al., 2008), including for alcohol (Chaudhri et al., 2010; Marchant et al., 2015), although, under conditions of extinction, inhibiting the mNAsh promotes relapse for alcohol (Millan et al., 2010). However, cortical activation of the mNAsh promotes reinstatement of opiate conditioned place preference (CPP) (Hearing et al., 2016) and seeking (Bossert et al., 2015), and OXRs within the mNAsh also contribute to expression and reinstatement of morphine CPP (Qi et al., 2013; Sadeghzadeh et al., 2016). Thus, although the mNAsh contribution can vary depending on the addictive behavior, these studies overall concur with our findings that OX1Rs in the mNAsh promote alcohol drinking *in vivo* and increase neuronal activity *ex vivo* (see below).

In contrast to the central role for mNAsh OX1Rs in driving alcohol consumption, OX1Rs within the aINS seemed to play no role in alcohol drinking. This was surprising because the aINS is thought to drive many addictive behaviors in both humans (Naqvi et al., 2014) and animals (Hollander et al., 2008; Seif et al., 2013), including compulsion-like alcohol drinking (Seif et al., 2013), and OX1Rs within the aINS mediate nicotine intake (Hollander et al., 2008). Thus, inasmuch as the aINS can promote alcohol drinking, it is likely that signaling systems other than OX1Rs within the aINS are required to promote addictive behavior. Also, OX1Rs in the medial prefrontal cortex promote cued reinstatement for alcohol in genetically-selected alcohol-preferring rats (Brown R. M. et al., 2015). In agreement, we found that mPFC OX1Rs were also important for promoting alcohol intake. Thus, our studies have identified critical regions of the cortico-accumbens circuit where OX1Rs act to promote excessive alcohol drinking.

Since the mNAsh can regulate feeding, including OXRs within the mNAsh (Thorpe and Kotz, 2005; Urstadt and Stanley, 2015; but see Baldo and Kelley, 2001), the reduction of alcohol consumption after inhibiting mNAsh OX1Rs could reflect more general effects on motor and consumption. However, intra-mNAsh OX1R inhibition had no effect on saccharin consumption or concurrent water intake during alcohol-drinking sessions, suggesting that mNAsh OX1Rs play a more specific role in driving alcohol consumption. In agreement, previous studies found no change in chow intake or locomotor activity when OX1Rs were inhibited in the mNAsh (Thorpe and Kotz, 2005; Kotani et al., 2008; Qi et al., 2013). In addition, several studies implicating the mNAsh in alcohol drinking also observed no reduction in intake of sweet substances (Stratford and Wirtshafter, 2011; Rewal et al., 2012; Kasten and Boehm, 2014; Lum et al., 2014). Thus, although there was a trend for a decrease in saccharin intake in our results with intra-mNAsh SB infusion, it is more likely that this does not reflect an actual but underpowered decrease. Also, we found that mNAsh OX1R inhibition significantly reduced alcohol drinking but not preference, which likely reflects the low level of concurrent water intake during the alcohol access session (a similar pattern is also observed in Dhaher et al., 2009; Seif et al., 2015; den Hartog et al., 2016). Thus, while mNAsh signaling can contribute to many consummatory behaviors, our findings suggest that OX1Rs within the mNAsh play a particular role in promoting excessive alcohol drinking.

In agreement with our observations that OX1R mNAsh promoted excessive alcohol drinking, we found that the orexin *ex vivo* enhanced action potential firing in mNAsh neurons from alcohol-drinking mice, and that this orexin increase in activity required OX1Rs. In contrast, there were no changes in evoked glutamatergic EPSCs, perhaps suggesting a primarily postsynaptic effect of orexin excitation within the mNAsh. In addition, orexin increased evoked firing with no changes in input resistance at the resting membrane potential. Interestingly, enhancement in mNAsh firing without changes at the hyperpolarized resting potential is similar to what has been reported for dopamine enhancement of mNAsh firing (Hopf et al., 2003). In agreement, NAc dopamine receptors regulate alcohol drinking and seeking (Bahi and Dreyer, 2012; Hauser et al., 2015), and dopamine receptors have been shown to interact with orexin to enhance mNAsh firing *ex vivo* (Mori et al., 2011). However, previous studies of orexin enhancement of mNAsh firing suggest a role for OX2Rs (Mukai et al., 2009; Mori et al., 2011), although these were performed in very young (12–16 d) animals, while our work was performed in adult neurons. Also, we did not find a role for OX2Rs within the mNAsh in promoting alcohol drinking. However, other work suggests that OX2Rs can interact with OX1Rs under some conditions, e.g., where either OX1R or OX2R inhibition in the mNAsh can suppress reinstatement of morphine CPP (Qi et al., 2013). Also, orexin enhancement of locomotion may be related to OX2Rs but not OX1Rs in the mNAsh (Thorpe and Kotz, 2005; Kotani et al., 2008), suggesting that mNAsh OX1Rs and OX2Rs can have differential effects. Furthermore, passive high alcohol exposures produce plasticity within the NAc Shell (Renteria et al., 2016), but we found that orexin enhanced firing *ex vivo* without changes in input resistance in both alcohol-drinking and alcohol-naïve mice. Nonetheless, understanding the *ex vivo* impact of orexinA is most critical in alcohol drinkers, since this physiological impact would be more relevant to the ability of mNAsh OX1Rs to drive alcohol intake.

It is also important to understand whether the ability of OX1Rs to enhance mNAsh postsynaptic firing, but not glutamatergic activation, has a more general implication for behavioral regulation by the mNAsh. Our observation that OX1Rs increased mNAsh activity and promoted alcohol drinking are in overall agreement with previous studies using agents that inhibit neuronal function, such as GABA receptor agonists, where mNAsh inhibition decreases alcohol drinking (e.g., Stratford and Wirtshafter, 2011; Kasten and Boehm, 2014; Ramaker et al., 2015). While these studies do not demonstrate the postsynaptic function *per se* is the primary site of action, it is very likely that the behavioral impact of these compounds reflects inhibition of the NAc neuron activity that is known to promote behavioral expression in a number of other paradigms (e.g., McGinty et al., 2013; Pascoli et al., 2014). Thus, any action of orexin (or other neurochemicals) that increases activity of NAc neurons could promote behavioral expression of alcohol drinking. In addition, enhancement of mNAsh firing or glutamatergic activity could increase excitatory throughput of the mNAsh and increase behavioral expression, especially since NAc neurons postsynaptic firing is strongly dependent on glutamatergic excitation (Gerfen and Surmeier, 2011). Thus, while modulation of specific glutamatergic inputs to the mNAsh could alter specific aspects of behavior (e.g., Pascoli et al., 2014), it remains unclear what selective effects that overall modulation of firing vs. glutamatergic activation would have. Finally, we found that mNAsh OX1Rs did not significantly regulate intake of saccharin, in agreement with other work finding no reduction in intake of sweet substances after mNAsh inhibition (see above). In fact, increased intake of sweet and other highly palatable substances has been associated with strong inhibition of the mNAsh rather than excitation (Richard et al., 2013). In contrast, mNAsh OXRs have been associated with increased food intake in some, although not all, studies (Baldo and Kelley, 2001; Thorpe and Kotz, 2005; Urstadt and Stanley, 2015). Thus, the neuronal pathway activated by mNAsh OX1Rs seems to play a more selective impact on intake of certain substances (alcohol, food), with perhaps a very different role for other substances (sweets); this might provide insight into the specific consummatory pathways co-opted by drives for alcohol.

It is important to note there have been mixed results regarding whether there are OX1Rs present in the NAshell and striatum, and that the OX1R-related blocker SB can still impact OX2Rs, although at lower affinity relative to OX1Rs (Smart et al., 2001). Importantly, however, our intracranial pharmacology experiments directly demonstrate that inhibition of OX1Rs but not OX2Rs within the mNAsh significantly reduced excessive alcohol drinking. These suggest that OX1Rs but not OX2Rs within the mNAsh promote excessive drinking. Nonetheless, while some studies have found some OX1R mRNA signal within the medial NAc (D'Almeida et al., 2005) and striatum (Hervieu et al., 2001, NAc not tested), others did not (Trivedi et al., 1998; Marcus et al., 2001). Also, a recent study utilizing a transgenic OX1R-GFP mouse found no OX1R-containing neurons within the NAc (Ch'ng and Lawrence, 2015). We note that mRNA levels do not always predict functional protein expression (e.g., as observed for OX1Rs for some brain regions described in Table 1 of Ch'ng and Lawrence, 2015). Also, small levels of receptor expression may be sufficient to allow functional signaling. e.g., where overlap of DA1R-cell and DA2R-cell markers can be seen in at least 20% of striatal neurons by electrophysiology and the very sensitive single-cell RT-PCR (Surmeier et al., 1996), even though transgenic GFP-expressing lines show very little overlap of DA1R-cell and D2R-cell markers in striatal neurons (Gerfen and Surmeier, 2011). In addition, OxA infusion within the mNAsh enhances feeding, which is prevented by SB, while OxA enhancement of locomotion is not prevented by SB (Thorpe and Kotz, 2005), indicating that SB can impact some but not all effects of OxA within the mNAsh, the remainder presumably reflecting action through OX2Rs. Also, development of morphine CPP is inhibited by SB but not OX2R inhibitors in the mNAsh (Sadeghzadeh et al., 2016). Finally, other studies have provided *ex vivo* evidence for functional OX1Rs within the mNAsh (Patyal et al., 2012), in addition to our demonstration of OX1R-dependent enhancement of mNAsh firing; we believe that these electrophysiological measures provide the most sensitive and direct method to assess the presence of functional receptors, in this case OX1Rs. Thus, since we directly demonstrated that inhibition of mNAsh OX2Rs with TCS had no impact on excessive alcohol drinking, our results taken together suggest that SB acts at OX1Rs within the mNAsh to suppress alcohol drinking. In addition, it is clear that the ability of OxR signaling to suppress alcohol drinking is region-specific, since both Insula and regions dorsal to the NAshell have Ox1Rs and OX2Rs, but SB within these regions had no impact on alcohol drinking. Finally, although our rat and mouse results implicate OX1R signaling within the mNAsh in promoting excessive alcohol drinking, we cannot completely rule out the possibility that the impact of intra-cranially infused SB inhibited alcohol drinking by action in a region medial or lateral to the mNAsh, such as the septum or NAc core. This is primarily a concern in mice, since we have previously shown that intra-mNAsh infusion in rats does not lead to effects within the adjacent NAc core, suggesting minimal effects of diffusion (Hopf et al., 2010). Also, the septum contains Ox1Rs although not mRNA for OX1Rs (see Wu et al., 2002; Ch'ng and Lawrence, 2015), while addiction-related orexin signaling has been associated with OX2Rs but not OX1Rs in the septum (Flores et al., 2016), and OX2Rs in the NAc core can regulate alcohol-related behavior (Brown et al., 2013). In contrast, here we found that OX1Rs but not OX2Rs drove excessive alcohol drinking in mice. Further experiments, perhaps involving local infusion of inhibitory RNAs, will be required to fully dissociate whether areas adjacent to the mNAsh are important for regulating alcohol drinking.

Taken together, our studies indicate that OX1Rs within the mNAsh are critical for promoting excessive alcohol drinking, which may reflect the ability of orexin to increase action potential firing in an OX1R-dependent manner in mNAsh neurons from alcohol drinkers. In contrast, OX1Rs within the aINS did not regulate alcohol consumption, even though the aINS can regulate alcohol intake (Seif et al., 2013) and aINS OX1Rs can promote nicotine intake (Hollander et al., 2008). Since OX1Rs play a predominant role in driving motivated intake (see above), and excessive drinking in humans is driven by pathological drives for alcohol, our results suggest that the mNAsh is a key region where OX1Rs can promote excessive alcohol intake. Thus, OX1R inhibitors might represent a viable therapeutic intervention to suppress alcohol drinking in humans (Khoo and Brown, 2014; Li et al., 2016).

## Author contributions

Study concept and design: KL, SW, FH. Acquisition of data: KL, SW, JY, AM, BH, FH; Statistical analyses and interpretation of data: KL, SW, FH; Preparation of manuscript: KL, FH.

## Funding

Supported by NIAAA P50 AA017072.

### Conflict of interest statement

The authors declare that the research was conducted in the absence of any commercial or financial relationships that could be construed as a potential conflict of interest.
